# Loss of Blood in Surgical Operations

**Published:** 1878-12

**Authors:** Alfred Fuchs

**Affiliations:** Berlin.


### ARTICLE VI.

### *Loss of Blood in Surgical Operations.*

Read before the American Dental Society of Europe.

BY ALFRED FUCHS, D. D. S., BERLIN.

Mr. President,—I would like to say a *few words* about a new method in *surgical* operations—of operations without a loss of blood. This subject, though not *directly* touching our restricted department of dental operations, must yet be of a certain interest to everyone here present, and might even eventually be applicable to certain cases of our own specialty.

The plan is to isolate, by a series of transverse ligatures, the part to be operated on; and the inventor, Dr. Langurbuch, of Berlin, claims that he can cut away for instance the lower (or upper lip,) or a part of the tongue, without the loss *of a drop of blood.*

The advantage of this is obvious. If the patient can be safely and deeply chloroformed, without fear of blood flowing into the larynx, the operation can be performed without undue haste and with a *certainty* of having taken away *all* the effected parts. The patient is not weakened by loss of blood.

Operations of the *tongue* were especially difficult on account of the abundant bleeding, which also makes it almost impossible to ascertain if the affected part has been *entirely* excored. By this novel method of procedure now these difficulties are as entirely done away with, as is the possibility of fillings getting wet since the invention and universal application of rubber dams.

The method of procedure will be best illustrated by a case in practice. Patient, a girl nine years of age, suffering of a cancerous tumor on the back of the tongue, which burst from time to time and bled profusely, so much so that the child's life was endangered.

The patient was chloroformed deeply, and the lower jaw lacerated to bring the posterior part of the tongue well forward and in view—the tongue is then grasped with a cross-forceps (by an assistant) and dragged as far as possible out of the mouth. A large curved needle, armed with ligature silk, is bored into the upper surface, right in the centre of the tongue, and pushed right through—two or three centimetres behind the tumor to be extirpated—the point of the needle is then turned towards the lower jaw (at a right angle to the tongue,) and the needle carried through the mucous membrane of the floor of the mouth back into the mouth one-half of the tongue, and the lingual artery running along the lower border of the tongue is thus secured. The same thing is then done with the other half of the tongue. The ligatures are then drawn together very tightly and knotted, and the long ends hanging out of the mouth serve to hold the tongue in position.

The tumor was excored, a few drops of blood escaped from it—not a drop from the part of the tongue left. The wound was sewn together, and the ligatures cut and drawn away.—*Dental Miscel any.*

